# The MEDEA childhood asthma study design for mitigation of desert dust health effects: implementation of novel methods for assessment of air pollution exposure and lessons learned

**DOI:** 10.1186/s12887-020-02472-4

**Published:** 2021-01-06

**Authors:** Panayiotis Kouis, Stefania I. Papatheodorou, Maria G. Kakkoura, Nicos Middleton, Emmanuel Galanakis, Eleni Michaelidi, Souzana Achilleos, Nikolaos Mihalopoulos, Marina Neophytou, Gerasimos Stamatelatos, Christos Kaniklides, Efstathios Revvas, Filippos Tymvios, Chrysanthos Savvides, Petros Koutrakis, Panayiotis K. Yiallouros

**Affiliations:** 1 Respiratory Physiology Laboratory, Medical School, University of Cyprus, Nicosia, Cyprus; 2 Shiakolas Educational Center of Clinical Medicine, Palaios Dromos Lefkosias-Lemesou 215/6, 2029 Aglantzia, Nicosia Cyprus; 3 Cyprus International Institute for Environmental & Public Health, Cyprus University of Technology, Limassol, Cyprus; 4 Department of Epidemiology, Harvard T.H. Chan School of Public Health, Harvard University, Boston, MA USA; 5 Clinical Trial Service Unit and Epidemiological Studies Unit CTSU, Nuffield Department of Population Health, University of Oxford, Oxford, UK; 6 Department of Nursing, Cyprus University of Technology, Limassol, Cyprus; 7 Medical School, University of Crete, Heraklion, Crete Greece; 8 Department of Chemistry, University of Crete, Heraklion, Greece; 9 Department of Civil & Environmental Engineering, University of Cyprus, Nicosia, Cyprus; 10 E.n.A Consulting LP, Arachova Boeotia, Greece; 11 Cyprus Broadcasting Corporation, Nicosia, Cyprus; 12 Department of Meteorology, Ministry of Agriculture, Rural Development and Environment, Nicosia, Cyprus; 13 Department of Labor Inspection, Ministry of Labor, Welfare and Social Insurance, Nicosia, Cyprus; 14 Department of Environmental Health, Harvard TH Chan School of Public Health, Boston, USA

**Keywords:** Desert dust, Asian dust, Asthma, Children, Public health intervention

## Abstract

**Background:**

Desert dust events in Mediterranean countries, originating mostly from the Sahara and Arabian deserts, have been linked to climate change and are associated with significant increase in mortality and hospital admissions from respiratory causes. The MEDEA clinical intervention study in children with asthma is funded by EU LIFE+ program to evaluate the efficacy of recommendations aiming to reduce exposure to desert dust and related health effects.

**Methods:**

This paper describes the design, methods, and challenges of the MEDEA childhood asthma study, which is performed in two highly exposed regions of the Eastern Mediterranean: Cyprus and Greece-Crete. Eligible children are recruited using screening surveys performed at primary schools and are randomized to three parallel intervention groups: a) no intervention for desert dust events, b) interventions for outdoor exposure reduction, and c) interventions for both outdoor and indoor exposure reduction. At baseline visits, participants are enrolled on MEDena® Health-Hub, which communicates, alerts and provides exposure reduction recommendations in anticipation of desert dust events. MEDEA employs novel environmental epidemiology and telemedicine methods including wearable GPS, actigraphy, health parameters sensors as well as indoor and outdoor air pollution samplers to assess study participants’ compliance to recommendations, air pollutant exposures in homes and schools, and disease related clinical outcomes.

**Discussion:**

The MEDEA study evaluates, for the first time, interventions aiming to reduce desert dust exposure and implement novel telemedicine methods in assessing clinical outcomes and personal compliance to recommendations. In Cyprus and Crete, during the first study period (February–May 2019), a total of 91 children participated in the trial while for the second study period (February–May 2020), another 120 children completed data collection. Recruitment for the third study period (February–May 2021) is underway. In this paper, we also present the unique challenges faced during the implementation of novel methodologies to reduce air pollution exposure in children. Engagement of families of asthmatic children, schools and local communities, is critical. Successful study completion will provide the knowledge for informed decision-making both at national and international level for mitigating the health effects of desert dust events in South-Eastern Europe.

**Trial registration:**

ClinicalTrials.gov: NCT03503812, April 20, 2018.

**Supplementary Information:**

The online version contains supplementary material available at 10.1186/s12887-020-02472-4.

## Background

### Desert dust storm events across southern Europe and human health

Mediterranean countries belong to the global dust belt, extending from West Africa to the Arabian Peninsula and populations residing in the region are frequently exposed to desert dust storms (DDS) [1]. During DDS events, particulate matter up to 10 μm (PM_10_) levels rise considerably higher above the EU daily limit value of 50 μg/m^3^ [2]. In the Eastern Mediterranean region, 10–15% of the days of the year are “DDS days”, while most of each year’s DDS events appear between February to May [2, 3]. Evidence from Eastern Mediterranean countries suggests that in the past 20 years there might have been an increase in the frequency and duration of DDS [2, 4, 5]. Climate change may be the driving factor for increased DDS frequency and duration, through: (1) an increase in desertification and desert surface temperature; (2) reduction in rainfall, which increases desertification and soil erosion and reduces PM washout from the atmosphere, and; (3) changes in the synoptic atmospheric patterns [6].

Desert dust particles are mostly composed of rock-forming and clay minerals but also carry microbial agents, such as bacteria, fungi and viruses [7], and absorb a mix of anthropogenic atmospheric pollutants during transport. Historically, DDS were not considered harmful to humans due to their natural origin and crustal composition. In line with this approach, EU legislation considers DDS impossible to prevent, implicitly harmless and discounts their contribution to daily and annual air quality standards of PM_10_. However, during the last two decades, several studies from around the world, as well as from Mediterranean countries such as Cyprus [8, 9] and Greece [10] have demonstrated associations of PM_10_ during DDS outbreaks with increased total and case-specific mortality and hospital admissions for asthma and chronic obstructive pulmonary disease.

The pathogenic effects of PM inhalation have been attributed to direct physical and toxic action of particles on human airway epithelium [11]. Exposure to DDS particles has also been associated to symptomatic exacerbations of pre-existing conditions reported as unscheduled hospital visits, use of excessive medication, loss of sense of well-being and days off work or school [12–14]. These relatively less severe consequences are more common, but largely unquantified and un-investigated. Children with asthma are considered one of the most vulnerable group to DDS exposure [10, 15, 16].

### Framework for designing, implementing and testing an adaptability strategy

Currently, during DDS events, EU national competent authorities and mass media in DDS-exposed regions issue non-standardised warnings to the public/vulnerable groups, most commonly advising them to stay indoors, and reduce outdoor activities. To date, no scientific evidence exists on the efficacy of any of these recommendations in either reducing exposure to DDS PM or mitigating related health effects.

### Study objectives

A demonstration project called “MEDEA” (Mitigating the Health Effects of Desert Dust Storms Using Exposure-Reduction Approaches) has been co-funded by the LIFE 2016 Programme (LIFE16 CCA/CY/000041) of the European Commission with the main goal to provide the field-based evidence for the feasibility and effectiveness of an adaptation strategy to DDS in South-Eastern Europe, focusing on exposure reduction approaches and inform EU policy making. The MEDEA Childhood Asthma panel study is implemented by seven partner institutions (see details in Supplementary Material) in highly DDS-exposed Mediterranean regions with the following specific objectives:

1. Design easy to implement and sustainable exposure-reduction recommendations to follow during DDS;

2. Demonstrate the feasibility of applying models for early forecasting of DDS events and timely notification of the public, targeting susceptible individuals, and;

3. Demonstrate which of the recommendations are effective in reducing exposure to DDS and disease-relevant adverse health effects in panels of vulnerable patients.

## Methods/design

### Overview of the project setting and design

To evaluate the comparative effectiveness of the recommendations, we have undertaken a clinical study in school-aged children with asthma in Cyprus and Crete. Patients (and schools) are randomized to three parallel intervention groups to receive: a) no additional intervention for DDS, b) project interventions for outdoor exposure reduction, and c) project interventions for both outdoor and indoor exposure reduction.

The MEDEA Asthma panel study has been registered with and approved by the clinicaltrials.gov online repository (ClinicalTrials.gov Identifier: NCT03503812) and national authorities at both sites (see details in Supplementary Material).

### Exposure reduction recommendations and the MEDena® health-hub

We have developed the recommendations for reduction of outdoor and indoor exposures to DDS and produced audiovisual spots for their implementation according to intervention group and audience (parents and teachers). As an example, animated guidelines in English addressed to parents participating in the outdoor exposure reduction intervention (Video S1) or the outdoor and indoor exposure reduction intervention (Video S2) are available as supplementary material. In brief, in outdoor intervention, participants are asked to stay indoors, as well as avoid intense physical activity outdoors, competitive sports and unnecessary walks. In indoor intervention, participants are asked to close windows and doors, seal possible cracks around windows and doors in order to minimize home ventilation, and use continuously an air cleaner in order to filter indoor air.


**Additional file 2: Video S1.**


**Additional file 3: Video S2.**

Concurrently, we have validated existing models for forecasting DDS events in Cyprus and Crete, and we have developed the MEDena® Health-Hub, a bidirectional, patient-centered web-based platform (Figure S1), and the MEDEApp® smart mobile application (Figure S2) for:

- collection of early forecasting data from meteorology modelers;
- early dissemination of warnings and audiovisual recommendations for exposure reduction to alert patients about upcoming DDS events;
- acquisition of accurate spatiotemporal activity and health data from patients via wearable sensors and online questionnaires, and;
- storage and management of personal exposure and health data with cloud technologies.

### Monitoring the intervention

On recruitment, participants are assigned an ID number and their personal, demographic, house and classroom location (coordinates) information are entered through the web to the MEDena® Health-Hub. Each patient wears a wristwatch and through the MEDEApp® mobile app installed in the Android smartphone of the parent the MEDena® Health-Hub tracks which patient ID is equipped with each device. The children, their parents and schoolteachers are trained in the tools and procedures to be followed and are given a leaflet with instructions on the use of the wristband and smartphone. Participants are asked to wear the smart wristbands 7 days a week, but not during sleep, bathing or swimming.

Following the eligibility assessment at the baseline visit, patients are assigned to the three parallel intervention groups at a 1:1:1 ratio. Assignment to the three parallel intervention groups is based on the randomization of their school to the three legs and interventions are implemented in the asthmatic child’s classroom and household settings. Details of monitoring the intervention can be found in Supplementary Material.

### Assessing compliance to intervention

**Fig. 1:**
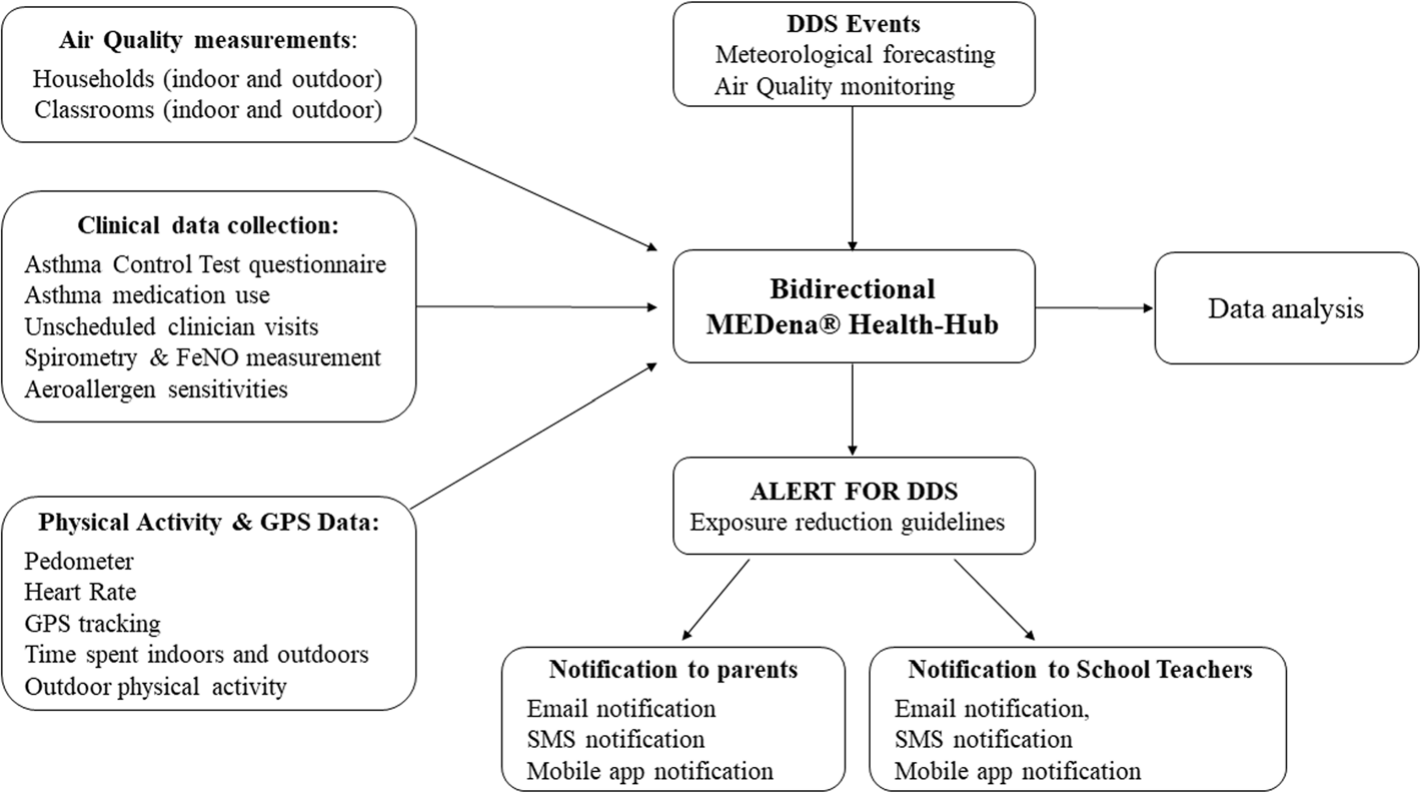
Asthma panel study schematic diagram. The bidirectional MEDena® Health-Hubis updated with meteorological forecasting and air-quality information regarding DDS events and sends alerts and exposure reduction guidelines to parents and teachers of asthmatic children participating in the study. At the same time, the MEDena® Health-Hub is automatically collecting the physical activity and GPS data from the wristbands worn by the children. Researchers also manually upload children clinical data and air quality measurements. DDS: Desert Dust Storm, FeNO: Fractional exhaled Nitric Oxide, GPS: Global Positioning System, SMS: Short Messaging Service text message

The participants’ behavioural patterns and compliance to the exposure-reduction guidelines are monitored in the three legs using wearable sensors, air pollution samplers and activity diaries. Compliance to outdoor recommendation to reduce time spent outdoors and avoid physical activity during DDS, is assessed continuously using a smart wristband (J-Style GPS watch tracker, model JC-1755, Joint Ltd., China), which is equipped with global positioning system (GPS) and activity tracking (pedometer) hardware and software. The wristband, continuously, collects the participant’s GPS and activity data and transmits them wirelessly to the MEDena® Health-Hub, a secure e-platform via Bluetooth connection to the MEDEApp® mobile app installed in the participants parents’ smartphone. Compliance to indoor recommendation to minimize home and classroom ventilation and use an air cleaner is assessed by using particle samplers (Harvard High Volume Cascade Impactors, Harvard University, USA) placed outside and inside representative participants’ houses and school classrooms and with indoor, commercial low volume air quality sensors (OPC-N3 Optical Particle Counters, Alphasense, United Kingdom). The Harvard samplers measure concentrations of PM_10_, PM_2.5_, black carbon (BC) and elements collected on Teflon filters at representative premises during DDS and DDS-free days. Alphasense sensors provide qualitative estimates of PM_10_ for a larger number of microenvironments in the three legs of the panel study. In addition, compliance to recommendations is assessed in all participants in the three legs through activity questionnaires following each DDS event. A schematic diagram of the Asthma panel study is presented in Fig. 1.

The GPS sensors continuously assess the time participants spend indoors and outdoors. For the time spent indoors, we assign PM_10_, PM_2.5_, BC and elements exposures measured by the indoor air pollutant samplers. For the time spent outdoors, we assign exposures to outdoor PM_10_ and PM_2.5_ levels as obtained from measurements of local air pollution monitoring stations and BC and elements measured by the outdoor samplers positioned outside representative premises.

### Project course and progress

The MEDEA project commenced in September 2017 and will continue through December 2022. During project year 1, we conducted pilot studies at both sites to test the feasibility of the set-up and protocols to assess exposures, activity and health outcomes in a small number of patients with and without implementation of exposure reduction recommendations.

During project years 2, 3 and 4, we perform the panel study in school-aged children with asthma and continue the assessment of the effectiveness of MEDEA recommendations to reduce indoor and outdoor exposure in the respective intervention legs of the studies. Simultaneously, we collect disease relevant health outcomes, as described below, to assess the effect of interventions.

In project year 5, we will demonstrate which of the recommendations are more effective to reduce exposure and associated adverse health effects in participating subjects. We will also address transferability of recommendations that are proven effective by targeting: a) citizens with social media and smartphone applications, and; b) institutions and competent EU authorities with the aim to assist the development of adaptation strategy in DDS-exposed South Europe areas.

### Panel study in schoolchildren with asthma in Cyprus and Crete (Greece)

#### Recruitment protocol - recruiting schools

In order to perform a challenging clinical trial like the MEDEA childhood asthma panel study that involves changes in behavior of children at the school and home environment, we pursue the highest collaboration and support from the schools’ authorities, as well as from the local teachers and parents’ communities. Details of recruitment of schools can be found in Supplementary Material. The International Study of Asthma and Allergies in Children, (ISAAC) questionnaire, available in both Greek and English, enriched with additional questions on medical care and medication utilization, is used to identify asthmatic children. Parents of the asthmatic children, who met our eligibility criteria, are contacted via telephone to inform them about the study and ask them to participate.

#### Population

**Fig. 2:**
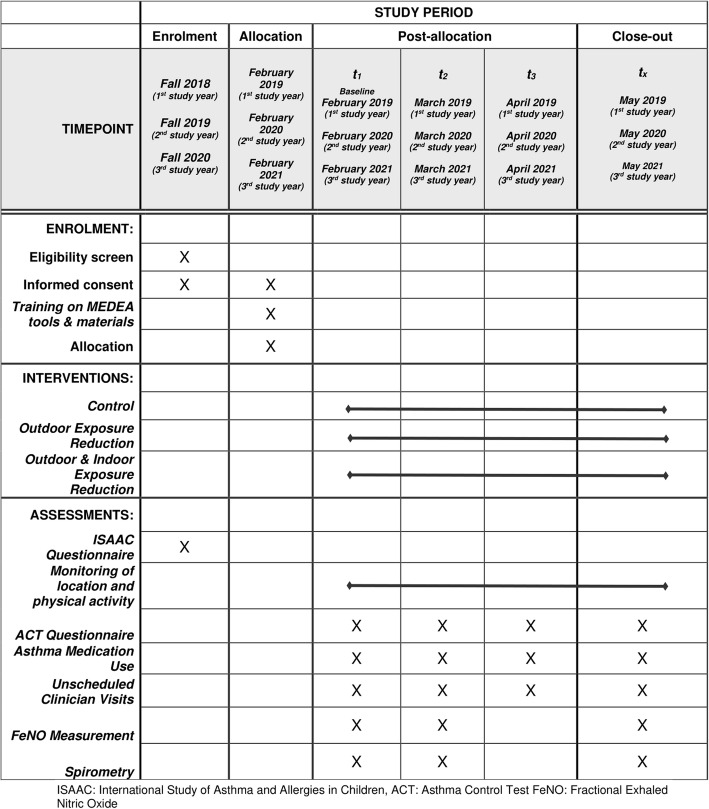
Childhood Asthma panel study SPIRIT flow diagram: The schedule of enrolment, interventions and assessments in the Childhood Asthma panel study according to SPIRIT template

The target population consists of children aged from 6 to 11 years from Greek- or English-speaking families who attend the participating schools in Cyprus and Crete during the academic years 2018–2019, 2019–2020 and 2020–2021. Children with mild to moderate persistent asthma are eligible for the study. Each child is assessed during only one high DDS period (February–May). The Standard Protocol Item: Recommendations for Interventional Trials (SPIRIT) flow diagram for the Asthma Panel study is presented in Fig. 2.

#### Definition of asthma

Children who report a physician’s diagnosis of asthma AND at least one of the following: wheezed in the past year, and/or, currently, take daily preventative asthma medication, and/or had an unscheduled medical visit for asthma in the past year, are considered eligible for the study. Exclusion criteria include lung disease other than asthma, cardiovascular disease or not living for at least 5 days per week in the same household.

#### Baseline and follow-up clinical assessments

Eligible asthmatic patients at screening are invited for a baseline clinical assessment in January 2019, 2020 and 2021 prior to the onset of the respective high DDS periods. During the baseline visit, questionnaires are administered to obtain data on socio-demographic characteristics, asthma and allergy symptoms, utilization of medical care and classroom and home environmental characteristics, including tobacco smoke exposure.

**Fig. 3:**
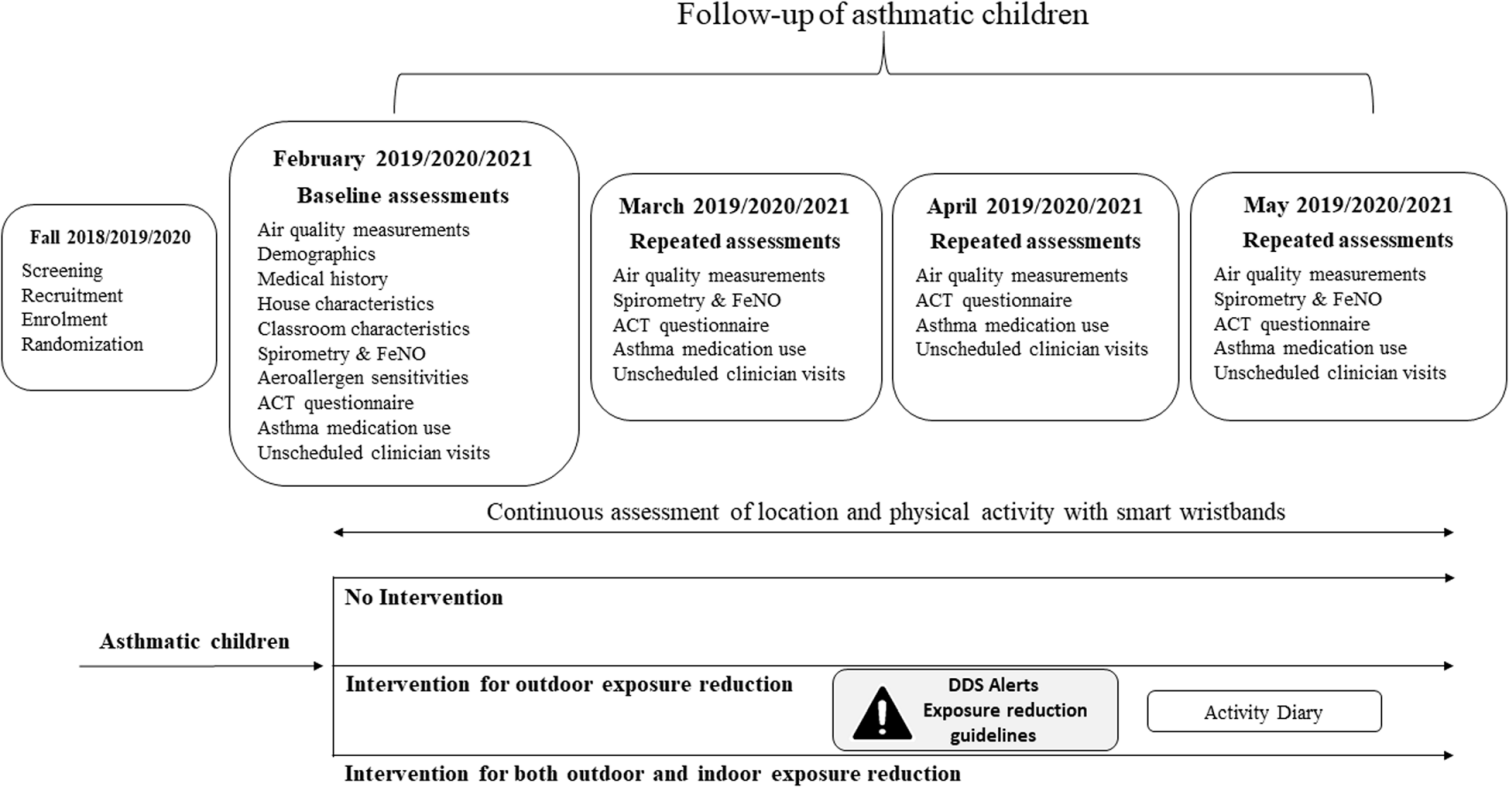
Childhood Asthma panel study assessments timeline. Timeline of baseline and follow-up assessments in Childhood Asthma panel study for the two study years. DDS: Desert Dust Storm, FeNO: Fractional exhaled Nitric Oxide, ACT: Asthma Control Test questionnaire

The follow-up period spans from February to late May/early June and includes continuous monitoring of the daily location and physical activity of patients using the wristbands and smartphones. Phone interviews at baseline and then at every 1 month throughout the high DDS period are performed collecting information on asthma symptoms control, medication use and unscheduled visits to health professional for asthma. Asthma control in the past 4 weeks is assessed using English and Greek versions of the pediatric Asthma Control Test (c-ACT, license number: QM044906) as used previously [17, 18]. Lastly, participants have assessments of lung function (Spirometry - In2itive Spirometer, Vitalograph Ltd., United Kingdom), Fractional exhaled nitric oxide (FeNO - NIOX VERO portable nitric oxide analyzer, Circassia, United Kingdom), at baseline, mid-period (April) and at the end of the follow up period (late May–June). At the end of the follow up period, skin prick testing to 14 common aero-allergens (Allergy Therapeutics PLC, United Kingdom) is performed as described previously [19]. The timeline and details of baseline and follow-up assessments for the asthma panel study are presented in Fig. 3 and in Supplementary Material.

#### Asthma morbidity outcomes and data analysis

The ACT questionnaire score is the primary health outcome and a change of 3 points in the total score is considered clinically meaningful [17]. For the primary analysis, we will compare the combined effect in the two intervention groups versus the control group. Next, we will compare between each of the intervention groups and control group and between the intervention groups. Secondary health outcomes will be the presence or absence of asthma symptoms in the prior 4-week period, asthma medication use, unscheduled visits for asthma, and values of forced expiratory volume in 1 s, peak expiratory flow and FeNO.

#### Sample size

Our primary health outcome, childhood ACT, has seven items and provides a score ranging from 0 (poorest asthma control) to 27 (optimal asthma control). A cut-off point of 19 indicates uncontrolled asthma [20] while previous studies have shown that the minimally meaningful change in the ACT score is 3 points [17]. To detect a statistically significant difference of 3 points, and assuming a 30% dropout rate, the minimum sample size needed in each of the 3 groups is 100 participants. This sample size calculation is assuming a level of 0.05 and a power of at least 80% to detect this difference between the comparison groups. In the first study period of February–May 2019, 91 children from six primary schools in Cyprus and eight primary schools in Crete participated in the clinical trial. For the second study period of February–May 2020, another 10 primary schools in Cyprus and 10 primary schools in Crete have been identified and additional 120 asthmatic children completed data collection. Recruitment for the third study period (February–May 2021) is underway.

#### Statistical analysis

Differences in the distribution of primary and secondary outcomes between the three intervention groups will be investigated using chi-square test for categorical parameters while the Wilcoxon Sum Rank test will be used for continuous parameters. In an adjusted analysis, the mean change in ACT score and other outcomes will be examined using a regression mixed effects model which will include fixed effects for intervention group and physical activity and subject-specific random intercepts and slopes for the control and intervention groups. The main spatio-temporal covariates that we will consider for the regression mixed-effects model are house characteristics, meteorological parameters (temperature, relative humidity) and local air pollution levels (PM_2.5_, PM_10_, and NOx). Additional details on the statistical analysis plan are available at: https://clinicaltrials.gov/ct2/show/NCT03503812.

## Discussion

The MEDEA Childhood Asthma panel study represents one of the largest, most comprehensive evaluation of the efficacy of recommendations aiming to reduce exposure to desert dust and related health effects in two highly exposed regions of the Eastern Mediterranean, Cyprus and Crete-Greece. In addition, the MEDEA Childhood Asthma panel study is unique because it benefits from the implementation of novel environmental epidemiology and telemedicine methods for assessing personal compliance to the recommendations, measuring exposure to air pollutants in home and school environments, and monitoring clinical outcomes among the study population.

The methods usually employed in air pollution health effects studies have several inherent inaccuracies in assessing exposure and health outcomes. Exposure estimates are commonly based on measurements conducted at monitoring stations that are sparsely distributed. These approaches use outdoor air pollution concentrations as a proxy for total exposure; therefore, they lack information on indoor air pollution levels, which introduces significant exposure error [21–23]. Furthermore, exposure estimates are usually assessed for a given residential address without taking into consideration participants’ activity and mobility throughout the day [24–26]. In MEDEA, we use wearable GPS and activity sensors and measure continuously activity and time asthmatic children spend indoors and outdoors. Thus, we are able to assign exposure to the respective air pollutants levels measured by indoor and outdoor samplers in representative premises, differentiated by the activity levels throughout the day providing a much higher spatiotemporal resolution. As a result, the uncertainties related to the variability of asthmatic children’s mobility during exposure assessment, are minimized and personal compliance and change in behaviour in response to the MEDEA recommendations can be assessed.

Health effects of desert dust exposure are usually assessed by using ecological retrospective data on major outcomes like deaths or hospital admissions and outpatient clinics’ visits [8–10, 27]. However, data on hospital admissions and outpatient clinics’ visits are influenced by subjective health care seeking behavior and are therefore, problematic in evaluation of the onset, duration and severity of an outcome [28]. In MEDEA Childhood Asthma panel study, we assess prospectively a range of clinical outcomes in both the control and interventions groups with standard clinical assessment tools like validated clinical symptoms questionnaires, lung function tests and other clinically relevant parameters.

During the first 2 years of the MEDEA project, we faced many unique challenges that we continue to address. The greatest challenge was overcome during the first year of the project. This is related to the development of the electronic sentinel platform (MEDena® Health-Hub), the selection and interface with the platform of adequate wearable devices and establishment of credible pathways of data acquisition from participants. Using smart devices requires a certain degree of technological literacy, which is challenging. In order to overcome this, we tested several commercially available smart watch devices. Subsequently, we chose the LEMFO-LM25 smartwatch equipped with embrace™ software (Embrace Tech LTD, Cyprus), which does not require manual synchronization with the created smartphone application to upload collected data but it does so automatically when it gets in contact with a WI-FI network. We also dealt with other limitations of smart devices such as missing data due to device malfunction, quick drain of wearable devices battery and need for frequent recharging, and reduced data storage issues. We were able to address these limitations by maintaining a very good and frequent communication with asthmatic children and their families in order to maintain their commitment and motivation to continue participation in the studies and not to forget wearing and frequently charging the smartwatch.

Performing environmental monitoring at homes and schools is challenging [29, 30]. Implementation of environmental interventions in schools and homes requires commitment by participating children, their families and schools. In order to maintain excellent engagement of the participants, our research staff monitored closely and follow-up our participating families not only by regular phone calls but also by paying regular visits, especially to the homes and classrooms of the participants in the combined outdoor-indoor intervention leg. During these visits, field workers reinforced implementation of recommendations for minimizing ventilation during DDS events, changing HEPA filters of the air cleaning devices, and ensuring that the air cleaners function effectively and are not inadvertently turned off.

A significant challenge is the analysis of GPS geo-spatial data and construction of individual participant’s trajectories for assessing personal exposures in outdoor and indoor microenvironments, which requires sophisticated algorithms [31, 32]. This is further complicated by an important limitation of GPS tracking, which is the loss of signal, especially in indoor environments, introducing the challenge of how to treat missing values questions. Automated microenvironment classification algorithms that include spatial and temporal buffering have been developed and validated, especially for air pollution exposure studies [33]. In an effort to have additional independent data on compliance besides the wearable sensors data, we also collect activity data after each DDS event with the use of a short-term recall questionnaire. Although replies to the questionnaire are still subjective, the short time frame between activity and questionnaire administration (few days to a maximum of 1 week), limits the potential recall bias and allows the assessment of compliance to the provided recommendations [34, 35].

## Conclusion

In MEDEA Childhood Asthma panel study, we evaluate for the first time interventions aiming to reduce exposure to DDS and implement novel environmental epidemiology and telemedicine methods in assessing personal compliance to the recommendations and related clinical outcomes. This requires the engagement of the entire local communities at each study site, asthmatic children, their families and schools. The commitment of large numbers of dedicated and talented researchers are also critical to the ongoing and continued success of this project. The successful completion of this study will provide the scientific knowledge for informed decision-making and strategic planning both at national (individual country stakeholders) and cross-national levels for mitigating the health effects of DDS events in South-Eastern Europe.

## Supplementary Information

**Additional file 1.**

## Data Availability

Not applicable.

## Abbreviations

DDS: Desert Dust Storms
PM_10_: Particulate Matter < 10 μm
PM_2.5_: Particulate Matter < 2.5 μm
GPS: Global Positioning system
BC: Black Carbon
ISAAC: International Study of Asthma and Allergy
ACT: Asthma Control Test
FeNO: Fractional Exhaled nitric oxide
