# Supplementary material for: The MEDEA childhood asthma study design for mitigation of desert dust health effects: implementation of novel methods for assessment of air pollution exposure and lessons learned
Source: BMC Pediatr. 2021 Jan 6;21:13. doi: 10.1186/s12887-020-02472-4 (PMC7786906; doi:10.1186/s12887-020-02472-4)
Supplement: Supplementary file 3 — Additional file 1. [file 12887_2020_2472_MOESM1_ESM.docx]

**Participating Institutions**

We have employed novel approaches to assess desert dust exposure and clinical outcomes implemented by an interdisciplinary consortium of seven institutions (University of Cyprus (Cyprus), Cyprus University of Technology (Cyprus), University of Crete (Greece), E.n.A Consulting LP (Greece), Cyprus Broadcasting Corporation (Cyprus), Department of Meteorology, Ministry of Agriculture, Rural Development and Environment (Cyprus) and Air Quality Sector, Department of Labour Inspection, Ministry of Labour, Welfare and Social Insurance (Cyprus) from Cyprus and Greece. The participating institutions were selected based on the following characteristics.

**i.** ability to adopt and adapt telemedicine practices to support air pollution intervention programs,

**ii.** prior knowledge of physical, chemical, meteorological and behavioral factors influencing exposures to particles, and;

**iii.** experience in performing epidemiological and risk assessment studies to evaluate the effectiveness of interventions in reducing human exposures and improving public health.

The final study dataset will be maintained by University of Cyprus and access will be provided to participating institutions based on the MEDEA Consortium Agreement on handling intellectual property issues.

**Funding Information**

This study was supported by the European Union LIFE project MEDEA (LIFE16 CCA/CY/000041). Additional funding information for this project are available at:

<https://ec.europa.eu/environment/life/project/Projects/index.cfm?fuseaction=search.dspPage&n_proj_id=6228&docType=pdf>

The funders had and will not have a role in study design, data collection and analysis, decision to publish, or preparation of this manuscript. Funder contact info: [easme-life@ec.europa.eu](mailto:easme-life@ec.europa.eu)

**Ethical approvals from national and institutional authorities**

The MEDEA Childhood Asthma panel study has been registered with and approved by the clinicaltrials.gov online repository (ClinicalTrials.gov Identifier: NCT03503812) and relevant authorities at both sites, according to national legislation. In Cyprus, approvals have been obtained from the Cyprus National Bioethics Committee (EEBK EΠ 2017.01.141), by the Data Protection Commissioner (No. 3.28.223) and Ministry of Education (No 7.15.01.23.5). In Greece, approvals have been obtained from the Scientific Committee (25/04/2018, No: 1748) and the Governing Board of the University General Hospital of Heraklion (25/22/08/2018). The interventions under study meet the criteria of the Helsinki declaration and follows the ICH/GCP and EC rules of good clinical practice. Data from individual countries are completely anonymized and encrypted prior to sending to the central database. Based on ethical approvals, access to the central database is limited to MEDEA personnel only.

**Methods supplementary information**

***Details of monitoring the intervention***

When predefined algorithms of PM_10_ levels are fulfilled, MEDEA air pollution scientists at each study site, promptly communicate through the e-platform alerts on the appearance of DDS to participants in the intervention legs of the panel study. To this effect, emails, smartphone applications and text messaging are used to disseminate exposure reduction recommendations in text and animated videos. The participants in both intervention groups have already been familiarised at recruitment with the respective intervention recommendations through the animated videos and take home hard-cover flyers printed in a user-friendly layout, to encourage commitment to the intervention. Similar leaflets detailing the intervention guidelines at schools have also been given at baseline to schools’ principals and teachers, so that they post them on their announcement boards.

In asthmatic children who are randomized to the combined outdoor and indoor intervention, we also arrange at recruitment to visit within the same week their houses and classrooms and install air-cleaning devices. Instructions on the use of air cleaners are provided to teachers and parents on site. During the home visit, we also assess the placement, and thus functionality of the air cleaner, in a room of the house where the child spends most of the time (typically between bedroom and sitting room). Reminders are taped on the air cleaners suggesting to keep them functioning continuously, throughout the four-month study period. The same process is also carried out at the classrooms of the asthmatic children and the air cleaning units are suggested to be continuously on as well. Principals and teaching staff are advised to do weekly checks of the air cleaners. Monthly clean-ups of the HEPA filters of the air cleaner are performed by the research staff, as recommended by the manufacturer.

***Details of recruiting schools & asthmatic children***

School principals, known from previous record to be supportive of child health promoting activities, are usually identified and contacted individually from the previous academic year and the details of the study are explained. At the beginning of the academic year, teaching staff and local parents associations of selected schools are invited to local meetings where the aims of the MEDEA project are presented and they are asked to partner our staff in a collaborative educational effort to promote personal health messages in the school and the community. In this direction, we have developed age-appropriate interactive presentations to schoolchildren on respiratory health, climatic change health effects, healthy diet and tobacco smoking effects. During fall, the presentations are delivered to children as part of the schools’ health education programs in order to connect with the students and teachers, and thus, try to engage them in the concepts and activities of MEDEA project.

In November-December following the interactive presentations, the International Study of Asthma and Allergies in Children, (ISAAC) questionnaire, available in both Greek and English, enriched with questions on medical care and medication utilization, is sent to the parents through their children. Instructions are given to students and their parents to fill out the questionnaire and return it to their teacher with the aim to detect eligible asthmatic children for participation in the study during the upcoming high DDS period of February-May. A small reward (a stationary ruler with the logo of the study) is also given to the students, as a measure to encourage the return of completed questionnaires.

***Baseline and follow-up clinical assessments of asthmatic children***

Asthmatic patients who fulfil inclusion/exclusion criteria at screening are invited for a baseline clinical assessment in January 2019, 2020 and 2021 prior to the onset of the respective high DDS periods. During the extensive baseline visit, informed consent is obtained, and the caretaker/child are asked detailed questions regarding socio-demographic characteristics, health symptoms, particularly asthma and allergy symptoms, and utilization of medical care, to further characterize the child’s asthma. A baseline questionnaire also assesses classroom and home environmental characteristics, including tobacco smoke exposure. Then participants have assessments of lung function (Spirometry - In2itive Spirometer, Vitalograph Ltd, United Kingdom), Fractional exhaled nitric oxide (FeNO - NIOX VERO portable nitric oxide analyser, Circassia, United Kingdom ), and skin prick testing to 14 common aero-allergens: olive (olea europea), tree pollens mix (Alder, Ash, Beech, Birch, Elm, Hazel, Oak, Plane, Poplar, Willow), cynodon dactylon, grass pollens mix (bent, brome, cocksfoot, dogstail meadow fescue, meadow foxtail, meadow grass, oat grass, rye grass, sweet vernal, timothy, yorkshire Fog), chenopodium album, salsola cali, alternaria alternata, cladosporium cladosporioides, aspergillus fumigatus, cat hair (felix catus), dog hair (canis familiaris), mouse hair (mus musculus), cockroach mixed, house dust mite (dermatophagoides pteronyssinus) (Allergy Therapeutics PLC, United Kingdom) as described before [1].

The follow-up period spans between February to late May-early June and includes continuous monitoring of the daily location and physical activity of patients using the wristbands and smartphones. Phone interviews at baseline and then at every 1 month throughout the high DDS period are performed collecting information on asthma symptoms control, medication use and unscheduled visits to health professional for asthma. Asthma control is assessed via caregiver and child’s responses to questions on daytime and night-time respiratory symptoms in the past 4 weeks, with the help of a validated Greek and English version of the pediatric Asthma Control Test (ACT) [2]. Lastly, at mid-period (April) and at the end of the follow up period (late May-June) MEDEA personnel visit the schools and reassess lung function and airway inflammation status (FeNO) of participating asthmatic children. The timeline of baseline and follow-up assessments in asthma panel study are summarized in Figure 3.

**References:**

1. Kolokotroni O, Middleton N, Gavatha M, Lamnisos D, Priftis KN, Yiallouros PK. Asthma and atopy in children born by caesarean section: effect modification by family history of allergies–a population based cross-sectional study. BMC pediatrics. 2012;12: 179.

2. Grammatopoulou EP, Skordilis EK, Stavrou N, Myrianthefs P, Karteroliotis K, Baltopoulos G, et al. The effect of physiotherapy-based breathing retraining on asthma control. Journal of Asthma. 2011;48: 593-601.

**Figure S1: MEDEA online platform design**


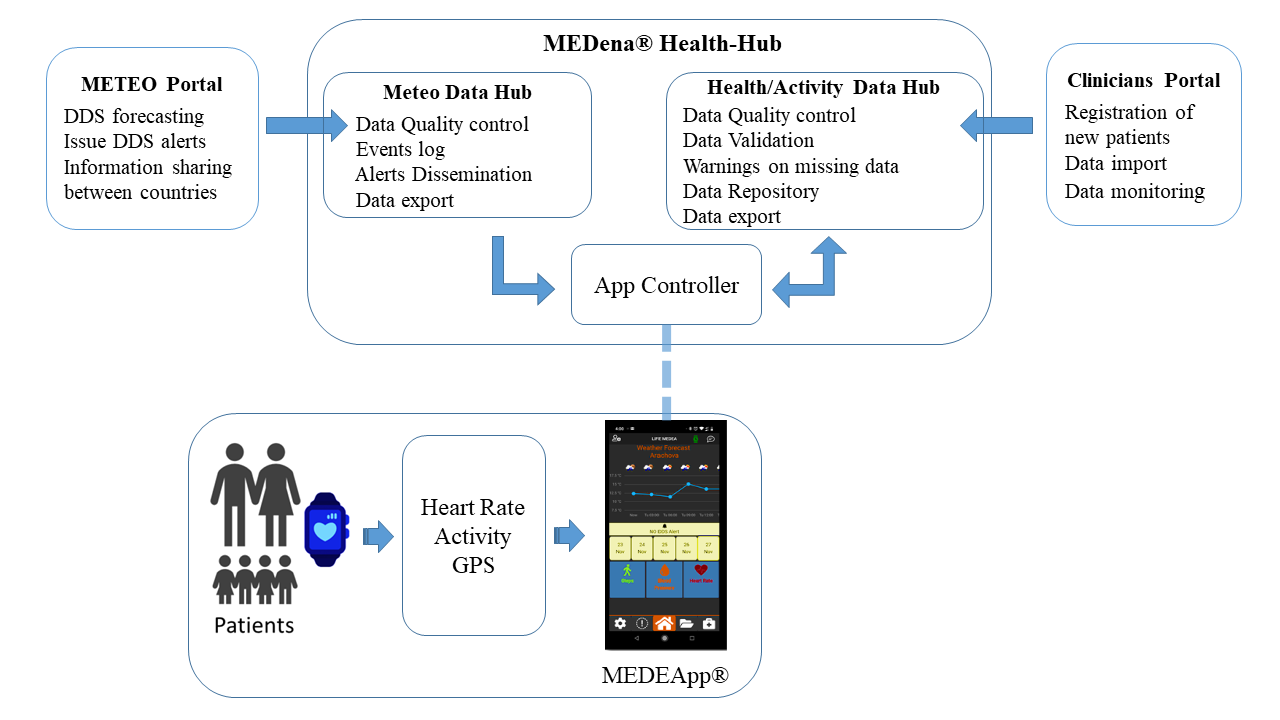


**Figure S1 Legend: MEDena® Health-Hub design.** The bidirectional MEDena® Health-Hub is accessible via the METEO and Clinicians portal. The Clinicians portal allow health professionals to register newly recruited patients and assign them to a particular intervention leg, study side and study period. Furthermore, it allows for the entry of questionnaire data as well as monitoring of accumulated data per participant. The METEO portal allows meteorologists and air quality experts to provide forecasting and issue alerts on DDS events that are disseminated to participants according to their respective intervention leg. The app controller allows for the real time communication between the MEDena® Health-Hub and the MEDEApp® mobile app and the synchronization of all collected activity data by the MEDEApp® mobile app with the Health/Activity Data Hub. Activity data are collected by the wristband and transferred via bluetooth to the MEDEApp® mobile app and automatically synchronised with the Health/Activity Data Hub through WiFi connection. A notable feature of the MEDena® Health-Hub is the ability to communicate warnings on missing data to both participants and researchers in the case that the participant has not synchronised his devise with the MEDena® Health-Hub for 24 hours. All communications between the MEDena® Health-Hub are carried out via SMS, email and app notifications.

**Figure S2: MEDEApp® mobile app overview**


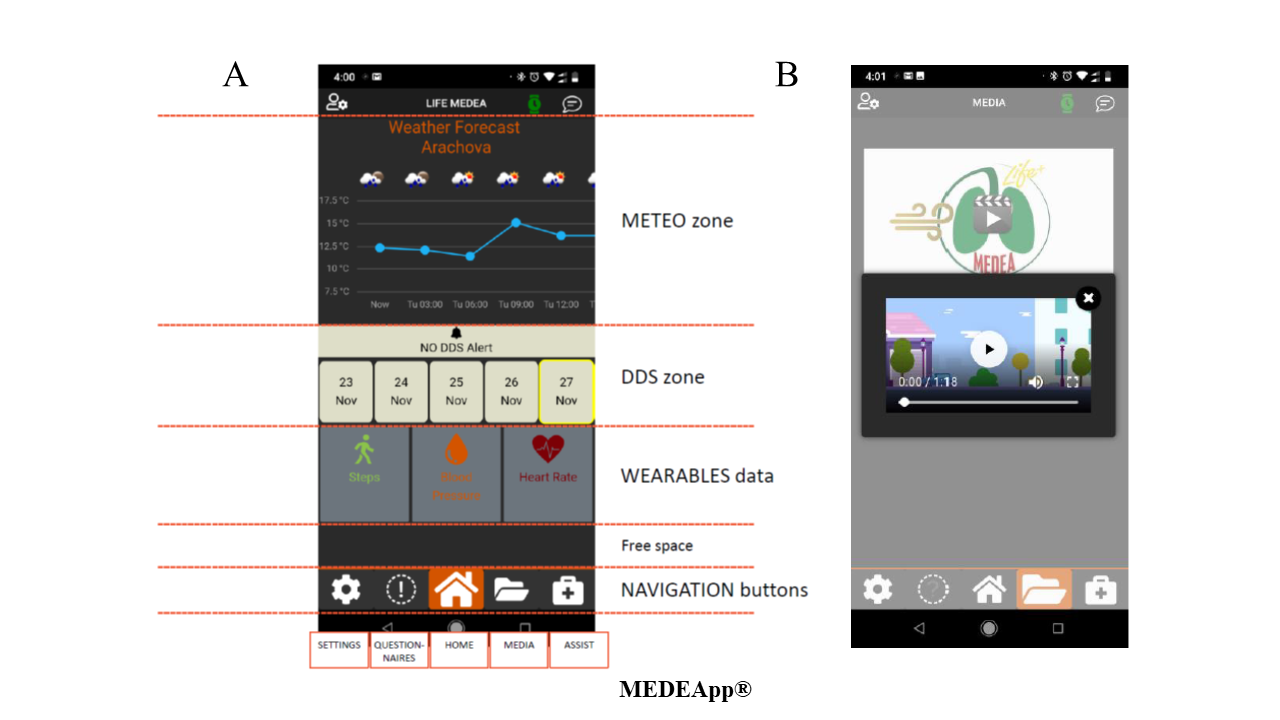


**Figure S2 legend: MEDEApp® mobile app overivew.** Panel A: The MEDEApp® mobile app features a user friendly design in an effort to maximise the usability of the application and maximise the engagement of the participant with its different features. It includes sections that provide information on the local weather and provide an overview of active or past DDS alerts as well as sections where the participants can also monitor their own data (heart rate, steps, calories, activity level). Panel B: In case of a DDS alert, a notification is displayed along with all the pre-defined exposure reduction guidelines and corresponding video with animated instructions according to each participant’s intervention leg. The video is also continuously available in the mobile application to further enhance training of the participant to the exposure reduction guidelines.

**Model Consent Form for Asthma Panel study (in Greek language)**

| **ΕΝΤΥΠΑ ΣΥΓΚΑΤΑΘΕΣΗΣ**  για συμμετοχή σε πρόγραμμα έρευνας  (Τα έντυπα αποτελούνται συνολικά από 4 σελίδες) |
| --- |

Καλείστε να συμμετάσχετε σε ένα ερευνητικό πρόγραμμα. Πιο κάτω (βλ. **«Πληροφορίες για Ασθενείς ή/και Εθελοντές»**) θα σας δοθούν εξηγήσεις σε απλή γλώσσα σχετικά με το τι θα ζητηθεί από εσάς και το παιδί σας ή/και τι θα σας συμβεί σε εσάς, εάν συμφωνήσετε να συμμετάσχετε στο πρόγραμμα. Θα σας περιγραφούν οποιοιδήποτε κίνδυνοι μπορεί να υπάρξουν ή ταλαιπωρία που τυχόν θα υποστείτε από τη συμμετοχή σας στο πρόγραμμα. Θα σας επεξηγηθεί με κάθε λεπτομέρεια τι θα ζητηθεί από εσάς και το παιδί σας και ποιος ή ποιοι θα έχουν πρόσβαση στις πληροφορίες ή/και άλλο υλικό που εθελοντικά θα δώσετε για το πρόγραμμα. Θα σας δοθεί η χρονική περίοδος για την οποία οι υπεύθυνοι του προγράμματος θα έχουν πρόσβαση στις πληροφορίες ή/και υλικό που θα δώσετε. Θα σας επεξηγηθεί τι ελπίζουμε να μάθουμε από το πρόγραμμα σαν αποτέλεσμα και της δικής σας συμμετοχής. Επίσης, θα σας δοθεί μία εκτίμηση για το όφελος που μπορεί να υπάρξει για τους ερευνητές ή/και χρηματοδότες αυτού του προγράμματος. **Δεν πρέπει να συμμετάσχετε, εάν δεν επιθυμείτε ή εάν έχετε οποιουσδήποτε ενδοιασμούς που αφορούν την συμμετοχή εσάς και του παιδιού σας στο πρόγραμμα.** Εάν αποφασίσετε να συμμετάσχετε, πρέπει να αναφέρετε εάν είχατε συμμετάσχει σε οποιοδήποτε άλλο πρόγραμμα έρευνας μέσα στους τελευταίους 12 μήνες. Εάν αποφασίσετε να μην συμμετάσχετε και το παιδί σας είναι ασθενής, η θεραπεία του δεν θα επηρεαστεί από την απόφασή σας. **Είστε ελεύθεροι να αποσύρετε οποιαδήποτε στιγμή εσείς επιθυμείτε την συγκατάθεση για την συμμετοχή εσάς και του παιδιού σας στο πρόγραμμα.** Εάν το παιδί σας είναι ασθενής, η απόφασή σας να αποσύρετε τη συγκατάθεση σας, δεν θα έχει οποιεσδήποτε επιπτώσεις στην θεραπεία του. Έχετε το δικαίωμα να υποβάλετε τυχόν παράπονα ή καταγγελίες, που αφορούν το πρόγραμμα στο οποίο συμμετέχετε, προς την Επιτροπή Βιοηθικής που ενέκρινε το πρόγραμμα ή ακόμη και στην Εθνική Επιτροπή Βιοηθικής Κύπρου.

Πρέπει όλες οι σελίδες των εντύπων συγκατάθεσης να φέρουν το ονοματεπώνυμο και την υπογραφή σας.

| Σύντομος Τίτλος του Προγράμματος στο οποίο καλείστε να συμμετάσχετε |
| --- |
| MEDEA  Mitigating the Health Effects of Desert Dust Storms Using Exposure-Reduction Approaches  Μείωση των Επιδράσεων των Καταιγίδων Σκόνης της Ερήμου στην Υγεία Υιοθετώντας Στρατηγικές Μείωσης της Έκθεσης |
| Υπεύθυνος του Προγράμματος στο οποίο καλείστε να συμμετάσχετε |
| Δρ Παναγιώτης Γιάλλουρος |

| Ονοματεπώνυμο **Πατέρα**: | |  | | |
| --- | --- | --- | --- | --- |
| Υπογραφή: |  | | Ημερομηνία: |  |

| Ονοματεπώνυμο **Μητέρας**: | |  | | |
| --- | --- | --- | --- | --- |
| Υπογραφή: |  | | Ημερομηνία: |  |

| Ονοματεπώνυμο **Ο έχων την γονική μεριμνα:** | |  | | |
| --- | --- | --- | --- | --- |
| Υπογραφή: |  | | Ημερομηνία: |  |

| Ονοματεπώνυμο **Συμμετέχοντα** : | |  | | |
| --- | --- | --- | --- | --- |
| Υπογραφή: |  | | Ημερομηνία: |  |

| **ΕΝΤΥΠΑ ΣΥΓΚΑΤΑΘΕΣΗΣ**  για συμμετοχή σε πρόγραμμα έρευνας  (Τα έντυπα αποτελούνται συνολικά από 4 σελίδες) |
| --- |
| Σύντομος Τίτλος του Προγράμματος στο οποίο καλείστε να συμμετάσχετε |
| Μείωση των Επιδράσεων των Καταιγίδων Σκόνης της Ερήμου στην Υγεία Υιοθετώντας Στρατηγικές Μείωσης της Έκθεσης |

| Δίδετε συγκατάθεση για τον εαυτό σας ή για κάποιο άλλο άτομο; |  |
| --- | --- |
| Εάν πιο πάνω απαντήσατε για κάποιον άλλο, τότε δώσετε λεπτομέρειες και το όνομα του. | |
|  | |

| Ερώτηση | ΝΑΙ ή ΟΧΙ |
| --- | --- |
| Συμπληρώσατε τα έντυπα συγκατάθεσης εσείς προσωπικά; |  |
| Τους τελευταίους 12 μήνες έχετε συμμετάσχει σε οποιοδήποτε άλλο ερευνητικό πρόγραμμα; |  |
| Διαβάσατε και καταλάβατε τις πληροφορίες για ασθενείς ή/και εθελοντές; |  |
| Είχατε την ευκαιρία να ρωτήσετε ερωτήσεις και να συζητήσετε το Πρόγραμμα; |  |
| Δόθηκαν ικανοποιητικές απαντήσεις και εξηγήσεις στα τυχόν ερωτήματά σας; |  |
| Καταλαβαίνετε ότι μπορείτε να αποσυρθείτε από το πρόγραμμα, όποτε θέλετε; |  |
| Καταλαβαίνετε ότι, εάν αποσυρθείτε, δεν είναι αναγκαίο να δώσετε οποιεσδήποτε εξηγήσεις για την απόφαση που πήρατε; |  |
| (Για ασθενείς) καταλαβαίνετε ότι, εάν αποσυρθείτε, δεν θα υπάρξουν επιπτώσεις στην τυχόν θεραπεία που παίρνει ή που μπορεί να πάρει το παιδί σας μελλοντικά; |  |
| **Συμφωνείτε να συμμετάσχετε εσείς και το παιδί σας στο πρόγραμμα;** |  |
| Με ποιόν υπεύθυνο μιλήσατε; | |

| Ονοματεπώνυμο **Πατέρα**: | |  | | |
| --- | --- | --- | --- | --- |
| Υπογραφή: |  | | Ημερομηνία: |  |

| Ονοματεπώνυμο **Μητέρας**: | |  | | |
| --- | --- | --- | --- | --- |
| Υπογραφή: |  | | Ημερομηνία: |  |

| Ονοματεπώνυμο **Ο έχων την γονική μεριμνα:** | |  | | |
| --- | --- | --- | --- | --- |
| Υπογραφή: |  | | Ημερομηνία: |  |

| Ονοματεπώνυμο **Συμμετέχοντα** : | |  | | |
| --- | --- | --- | --- | --- |
| Υπογραφή: |  | | Ημερομηνία: |  |

| **ΕΝΤΥΠΑ ΣΥΓΚΑΤΑΘΕΣΗΣ**  για συμμετοχή σε πρόγραμμα έρευνας  (Τα έντυπα αποτελούνται συνολικά από 4 σελίδες) |
| --- |
| Σύντομος Τίτλος του Προγράμματος στο οποίο καλείστε να συμμετάσχετε |
| Μείωση των Επιδράσεων των Καταιγίδων Σκόνης της Ερήμου στην Υγεία Υιοθετώντας Στρατηγικές Μείωσης της Έκθεσης |

**ΠΛΗΡΟΦΟΡΙΕΣ ΓΙΑ ΑΣΘΕΝΕΙΣ ή/και ΕΘΕΛΟΝΤΕΣ**

Εσείς και το παιδί σας καλείστε να συμμετάσχετε στην προοπτική μελέτη για τη μείωση της έκθεσης σε αυξημένες συγκεντρώσεις σωματιδίων σκόνης που παρατηρούνται κατά τα επεισόδια καταιγίδων σκόνης της ερήμου (ΚΣΕ) που διεξάγεται από το Πανεπιστήμιο Κύπρου στα πλαίσια του ευρωπαϊκού προγράμματος LIFE+ MEDEA. Με το παρόν έντυπο θα θέλαμε να σας ενημερώσουμε για τις λεπτομέρειες της μελέτης και να ζητήσουμε τη συμμετοχή σας, αφού πρώτα διαβάσετε με προσοχή τις παρακάτω πληροφορίες.

**ΣΚΟΠΟΣ ΤΗΣ ΜΕΛΕΤΗΣ**

Επιστημονικές μελέτες αρκετών χρόνων έχουν δείξει ότι τα επεισόδια ΚΣΕ στις χώρες της Ανατολικής Μεσογείου, προερχόμενα κυρίως από τις ερήμους της Σαχάρας και της Αραβικής Χερσονήσου, έχουν αυξηθεί σε αριθμό και ένταση. Τα επεισόδια ΚΣΕ είναι πολύ συχνά και στην Κύπρο και πλήθος επιδημιολογικών μελετών έχουν δείξει ότι συνδεόνται με αρνητικές επιπτώσεις στην υγεία, ιδίως σε υποομάδες του πληθυσμού με ιδιαίτερη ευαισθησία στους ατμοσφαιρικούς ρύπους, όπως τα ασθματικά παιδιά. Σκοπός του προγράμματος LIFE+ MEDEA είναι η ανάπτυξη εφαρμόσιμων και βιώσιμων οδηγιών για υιοθέτηση κατά τη διάρκεια των επεισοδίων ΚΣΕ με σκοπό τη μείωση της έκθεσης σε ατμοσφαιρικούς ρύπους που σχετίζονται με αυτά τα επεισόδια. Η αποτελεσματικότητα των οδηγιών αυτών για τη μείωση της έκθεσης σε αέριους ρύπους από ΚΣΕ και των επιδράσεων τους στην υγεία θα αξιολογηθεί κατά την διάρκεια Παρεμβατικής Μελέτης Δημόσιας Υγείας σε ομάδες ασθματικών παιδιών. Τα δεδομένα που θα συλλεχθούν από τους συμμετέχοντες θα γίνουν πλήρως ανώνυμα και θα τύχουν στατιστικής ανάλυσης για εξαγωγή αποτελεσμάτων. Απώτερος στόχος του προγράμματος, είναι οι οδηγίες που θα δοκιμαστούν στην συγκεκριμένη μελέτη να καταστούν εθνικές οδηγίες και συνήθης πρακτική σε όλες τις χώρες που παρουσιάζουν μεγάλη συχνότητα επεισοδίων ΚΣΕ.

**ΠΕΡΙΓΡΑΦΗ ΜΕΛΕΤΗΣ**

Τα ασθματικά παιδιά, είναι ιδιαίτερα ευαίσθητα στην αύξηση των επιπέδων σωματιδίων σκόνης από επεισόδια ΚΣΕ. Τα ασθματικά παιδιά που θα συμμετάσχουν στο πρόγραμμα θα χωριστούν με τυχαίο τρόπο σε τρείς ομάδες. Η μία ομάδα θα λαμβάνει ειδοποιήσεις για επερχόμενα επεισόδια ΚΣΕ και οδηγίες για το πως να μειώσει την έκθεση της σε σωματίδια σκόνης στους εξωτερικούς χώρους (σε κινητό τηλεφωνο – smartphone), η δεύτερη θα λαμβάνει ειδοποιήσεις για επερχόμενα επεισόδια ΚΣΕ για το πως να μειώσει την έκθεση της σε σωματίδια σκόνης στους εξωτερικούς και εσωτερικούς χώρους (σε κινητό τηλέφωνο – smartphone) ενώ μια ομάδα δεν θα λαμβάνει καθόλου ειδοποιήσεις και καθόλου οδηγίες.

| Ονοματεπώνυμο **Πατέρα**: | |  | | |
| --- | --- | --- | --- | --- |
| Υπογραφή: |  | | Ημερομηνία: |  |

| Ονοματεπώνυμο **Μητέρας**: | |  | | |
| --- | --- | --- | --- | --- |
| Υπογραφή: |  | | Ημερομηνία: |  |

| Ονοματεπώνυμο **Ο έχων την γονική μεριμνα:** | |  | | |
| --- | --- | --- | --- | --- |
| Υπογραφή: |  | | Ημερομηνία: |  |

| Ονοματεπώνυμο **Συμμετέχοντα** : | |  | | |
| --- | --- | --- | --- | --- |
| Υπογραφή: |  | | Ημερομηνία: |  |

| **ΕΝΤΥΠΑ ΣΥΓΚΑΤΑΘΕΣΗΣ**  για συμμετοχή σε πρόγραμμα έρευνας  (Τα έντυπα αποτελούνται συνολικά από 4 σελίδες) |
| --- |
| Σύντομος Τίτλος του Προγράμματος στο οποίο καλείστε να συμμετάσχετε |
| Μείωση των Επιδράσεων των Καταιγίδων Σκόνης της Ερήμου στην Υγεία Υιοθετώντας Στρατηγικές Μείωσης της Έκθεσης |

Και στις τρεις ομάδες παιδιών θα γίνεται αξιολόγηση της κατάστασης της υγείας τους από έμπειρους επαγγελματίες υγείας πριν, κατά τη διάρκεια και μετά την περίοδο όπου παρουσίαζεται μεγάλη συχνότητα επεισοδίων καταιγίδων σκόνης. Η αξιολόγηση της υγείας περιλαμβάνει συνδυασμό τηλεφωνικών συνεντεύξεων (χρήση Ερωτηματολογίου Ελέγχου του άσθματος – Αsthma Control Test), με καταγραφή των αλλαγών στη χρήση φαρμακευτικής αγωγής για το άσθμα και με μη επεμβατική μέτρηση της αναπνευστικής λειτουργίας (σπειρομετρία και μέτρηση εκπνεόμενου μονοξειδίου του αζώτου). Η συγκεκριμένη μελέτη δεν περιλαμβάνει οποιαδήποτε ιατροφαρμακευτική παρέμβαση στους συμμετέχοντες.

**ΠΛΗΡΟΦΟΡΙΕΣ ΓΙΑ ΤΗΝ ΑΣΦΑΛΕΙΑ ΤΩΝ ΔΕΔΟΜΕΝΩΝ**

Μερικά στοιχεία ταυτοποίησης πρέπει να καταχωρούνται κατά την εγγραφή του ασθενούς στην μελέτη, όπως ο μήνας και ο χρόνος γέννησης. Αυτά τα στοιχεία θα μεταφέρονται αμέσως στο σύστημα δημιουργίας κώδικα ταυτοποίησης (Patient identification-Code - PID) και αυτόματα θα δημιουργείται ψευδώνυμο (κωδικός) για τον κάθε ασθενή. Η ανακάλυψη της ταυτότητας του ασθενούς μέσω του PID θα είναι αδύνατη. Κάθε πληροφορία που σας αφορά θα είναι αυστηρά εμπιστευτική και προστατεύεται από τους κανόνες του ιατρικού απορρήτου καθώς και από την Εθνική Νομοθεσία για την προστασία των προσωπικών δεδομένων. Σε καμία από τις δημοσιεύσεις που αφορούν στη συγκεκριμένη μελέτη δεν θα χρησιμοποιηθούν προσωπικά δεδομένα.

**Με την υπογραφή σας παρακάτω δηλώνετε ότι συμφωνείτε να:**

- Λάβετε μέρος εσείς και το παιδί σας στην προοπτική μελέτη για τη μείωση της έκθεσης σε σωματίδια σκόνης που σχετίζονται με επεισόδια ΚΣΕ.
- Να γίνει χρήση των ανώνυμων δεδομένων σας για τους σκοπούς του παρόντος προγράμματος όπως αυτοί περιγράφονται στο τμήμα ‘Πληροφορίες για ασθενείς και εθελοντές’

Σε περίπτωση που έχετε οποιοδήποτε παράπονο σε σχέση με την έρευνα αυτή μπορείτε να επικοινωνήσετε με:

Α) Προϊστάμενο Υπηρεσίας Υποστήριξης Έρευνας Πανεπιστημίου Κύπρου, κ. Μάριο Δημητριάδη ([demetriades.a.marios@ucy.ac.cy](mailto:demetriades.a.marios@ucy.ac.cy))

Β) Κοσμήτορα της Ιατρικής Σχολής Πανεπιστημίου Κύπρου, Δρ Νικόλαο Παυλίδη ([pavlidis.nicholas@ucy.acy.cy](mailto:pavlidis.nicholas@ucy.acy.cy) )

| Ονοματεπώνυμο **Πατέρα**: | |  | | |
| --- | --- | --- | --- | --- |
| Υπογραφή: |  | | Ημερομηνία: |  |

| Ονοματεπώνυμο **Μητέρας**: | |  | | |
| --- | --- | --- | --- | --- |
| Υπογραφή: |  | | Ημερομηνία: |  |

| Ονοματεπώνυμο **Ο έχων την γονική μεριμνα:** | |  | | |
| --- | --- | --- | --- | --- |
| Υπογραφή: |  | | Ημερομηνία: |  |

| Ονοματεπώνυμο **Συμμετέχοντα** : | |  | | |
| --- | --- | --- | --- | --- |
| Υπογραφή: |  | | Ημερομηνία: |  |
